# Interactive Holographic Display Based on Finger Gestures

**DOI:** 10.1038/s41598-018-20454-6

**Published:** 2018-01-31

**Authors:** Shota Yamada, Takashi Kakue, Tomoyoshi Shimobaba, Tomoyoshi Ito

**Affiliations:** Graduate School of Engineering, Chiba University, 1-33 Yayoi-cho, Inage-ku, Chiba, 263-8522 Japan

## Abstract

In this paper, we demonstrate an interactive, finger-sensitive system which enables an observer to intuitively handle electro-holographic images in real time. In this system, a motion sensor detects finger gestures (swiping and pinching) and translates them into the rotation and enlargement/reduction of the holographic image, respectively. By parallelising the hologram calculation using a graphics processing unit, we realised the interactive handling of the holographic image in real time. In a demonstration of the system, we used a Leap Motion sensor and a phase modulation-type spatial light modulator with 1,920 × 1,080 pixels and a pixel pitch of 8.0 µm × 8.0 µm. The constructed interactive finger-sensitive system was able to rotate a holographic image composed of 4,096 point light sources using a swiping gesture and enlarge or reduce it using a pinching gesture in real time. The average calculation speed was 27.6 ms per hologram. Finally, we extended the constructed system to a full-colour reconstruction system that generates a more realistic three-dimensional image. The extended system successfully allowed the handling of a full-colour holographic image composed of 1,709 point light sources with a calculation speed of 22.6 ms per hologram.

## Introduction

Three-dimensional (3D) displays have attracted considerable attention in the digital signage^1,2^, entertainment^3^ and medical fields^4–6^. Holography^7,8^ is a 3D display technique that can display a natural 3D image close to an actual object and does not require scanning and synchronisation processing because it records and reconstructs light waves emitted from the object via light interference and diffraction. The interference between the light from the object and the reference light generates an interference fringe pattern called a hologram. Since the hologram is recorded on a photosensitive material, it is difficult to use classical holography to record and reconstruct motion pictures. Electro-holography^9^, which reconstructs 3D images using a spatial light modulator (SLM), was proposed to overcome the above shortcoming of classical holography. Thus, electro-holography can reconstruct 3D motion pictures by displaying computer-generated holograms (CGHs) obtained by simulating light propagation and interference on computers at each frame.

Although interactive 3D display systems that enable an observer to intuitively deal with 3D images have been developed based on fog screens^10^, rotating 2D displays^11^ or multiple 2D displays^12^, these systems require many projectors, multiple displays or high-speed mechanical processing to reconstruct natural 3D images. These systems also require synchronisation amongst all devices and a movie. Although many studies on electro-holographic displays^13–24^ have succeeded in real-time reconstruction using large-scale field-programmable gate arrays, graphics processing units (GPUs) and algorithms that speed up the calculation of CGHs, most of these studies realised only unidirectional systems that allow the observation but not the handling of the reconstructed images. On the other hand, interactive switching of CGHs with a motion sensor has been reported using optical tweezers^25,26^, which manipulate nano- and micron-sized particles in the contactless mode using a focused laser beam. Optical tweezers with CGHs (holographic optical tweezers) use CGHs to generate multiple focusing spots and can realise the interactive manipulation of small particles without the mechanical scanning process by switching the CGHs in real time. However, when using holographic optical tweezers, a manipulator does not observe the holographically reconstructed 3D images in real space but rather observes the two-dimensional (2D) images captured by a digital camera via a 2D display during the manipulations. This indicates that holographic optical tweezers have not realised the direct handling of holographic images.

**Figure 1:**
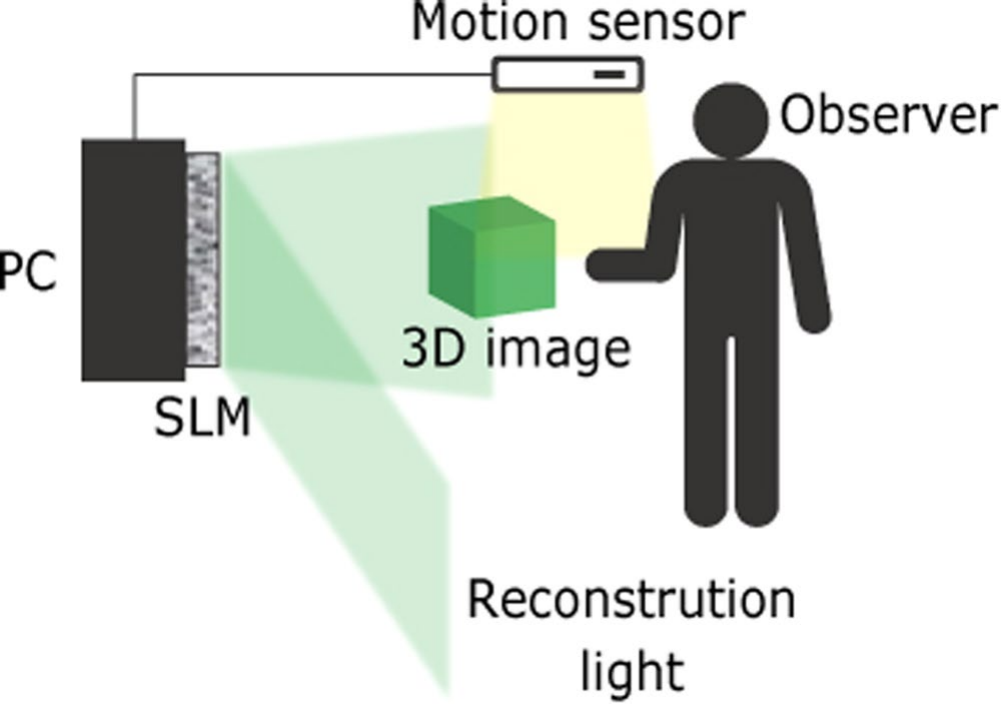
Schematic of the interactive handling system.

Thus, we aimed to construct a bidirectional electro-holographic system that allows real-time interaction with the reconstructed images. Although the light-field display^27,28^, which can also reconstruct 3D images, has been successfully used for the interactive handling of 3D images, it has a drawback: the resolution of the reconstructed 3D images decreases when the images are reconstructed away from the display plane. This results from the loss of the phase information of the light. Because the light-field technique is based on geometrical optics, it cannot record and reconstruct the phase information of the light. This is problematic because the phase information significantly contributes to the resolution of the reconstructed 3D images^29^. On the contrary, holography can record and reconstruct phase information because it is based on wave optics. The electro-holographic images generated by Yamaguchi’s interactive system^30^ can be controlled by touch. However, this system cannot generate and reconstruct the electro-holographic images in real time. We have demonstrated an interactive electro-holography system in which the 3D image can be operated in real time using a keyboard^31^. However, using the keyboard as an interface to manipulate the images is non-intuitive. Hence, we have constructed an interactive system in which the observer can intuitively handle the electro-holographic image in real time through hand and finger movements, which are detected by a motion sensor (Fig. 1). This intuitive control mechanism provides a realistic, immersive environment for the observer. This type of intuitive handling of 3D images will likely be required in virtual-reality holographic displays in the future. In this paper, we demonstrate an interactive finger-sensitive system which enables an observer to handle electro-holographic images in real time using intuitive finger gestures.

## Results

**Figure 2:**
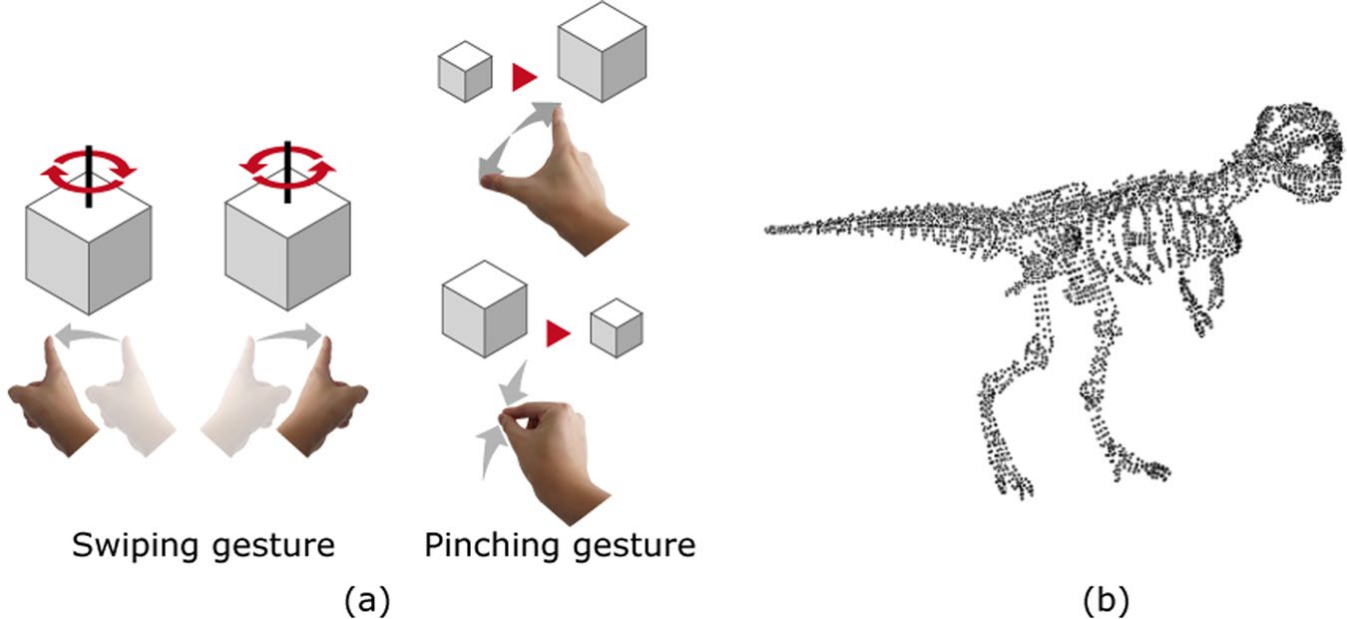
Schematics showing (**a**) the finger gestures adopted in this experiment for handling reconstructed images and (**b**) the 3D object used in this experiment. The 3D object consisted of 4,096 point light sources.

Figure 2a shows a schematic of the constructed system. First, the system detects the gestures of the observer using a motion sensor. Upon detection of a swiping gesture, the system rotates a 3D object composed of point clouds in a clockwise (leftward swipe) or anticlockwise (rightward swipe) direction. Here, the system performs the rotation process by multiplying the coordinate of each point by the rotation matrix. When the system detects a pinching gesture, the system enlarges (pinching out) or reduces (pinching in) the size of the 3D object. Here, the system performs the enlargement process by multiplying the coordinate of each point by the scale coefficient. Subsequently, the system calculates a CGH of the 3D object based on the rotation, enlargement or reduction operation. Finally, the system displays the CGH on the SLM and reconstructs a holographic 3D image by illuminating light to the SLM. The processing steps described above can be regarded as one frame of the electro-holographic image; thus, the interactive handling of electro-holographic motion pictures can be realised by repeating these processing steps. Figure 2b shows a schematic of the 3D object of a dinosaur composed of 4,096 point light sources. The 3D object was virtually placed 1 m away from the SLMs when calculating the CGHs.

**Figure 3:**
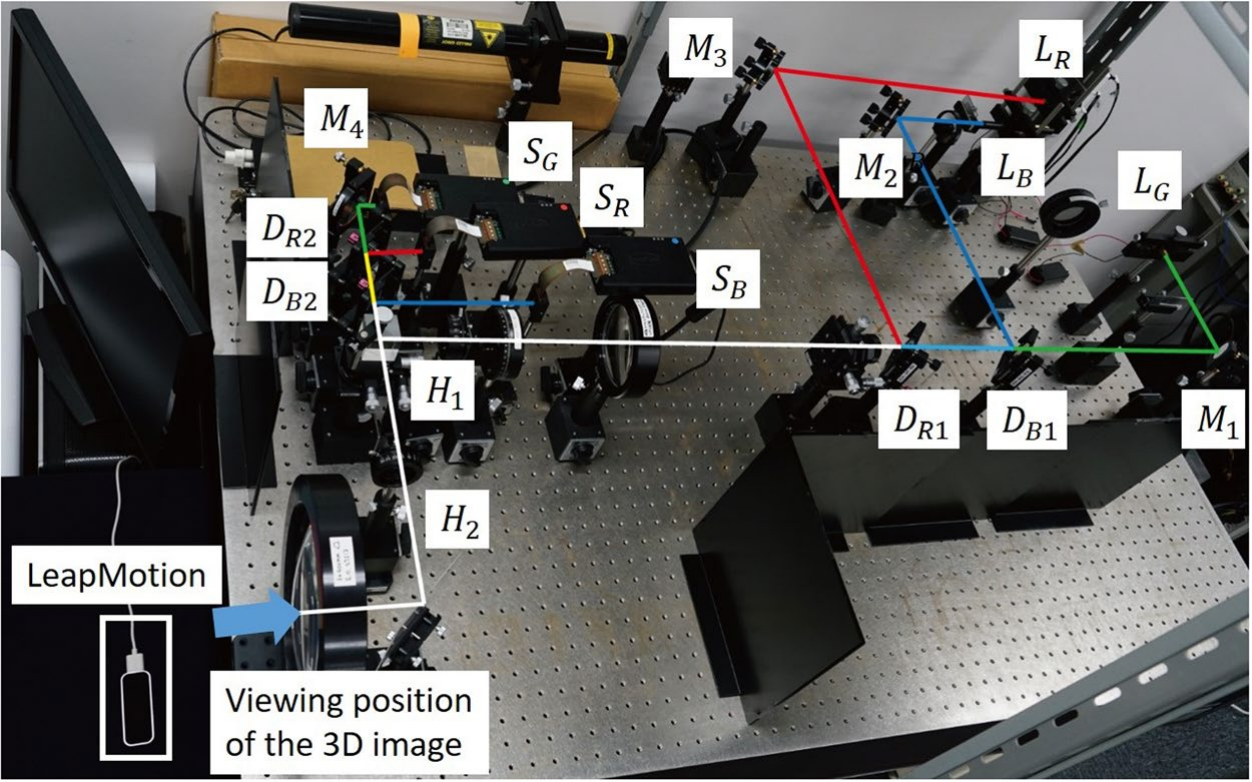
Experimental setup used for electro-holographic reconstruction.

Figure 3 shows the experimental setup used to demonstrate the full-colour electro-holography reconstruction system. First, we used only a green laser (532 nm) and reconstructed monochromatic holographic images. A phase modulation-type SLM (Holoeye Photonics AG, ‘PLUTO’) with 1,920 × 1,080 pixels and a pixel pitch of 8.0 µm × 8.0 µm was used to display the CGHs. The gradation of phase modulation and refresh rate of the SLM were 256 and 60 Hz, respectively. A Leap Motion (Leap Motion Inc.)^32^ motion sensor was used to detect finger gestures. This sensor is designed to detect motion in hands and fingers with high accuracy at more than 100 fps. A central processing unit (CPU; Intel Core i7-7700K with 4.2 GHz) and GPU (NVIDIA, ‘GeForce GTX1080Ti’) parallelised the processing of point clouds and calculation of CGHs.

*L*_*R*_, *L*_*G*_, *L*_*B*_: Red, green and blue lasers, respectively. *S*_*R*_, *S*_*G*_, *S*_*B*_: SLMs for red, green and blue reconstruction, respectively. *M*_1_~*M*_4_: Mirrors. *H*_1_, *H*_2_: Half mirrors. *D*_*B*1_, *D*_*B*2_, *D*_*R*1_, *D*_*R*2_: Dichroic mirrors.

**Figure 4:**
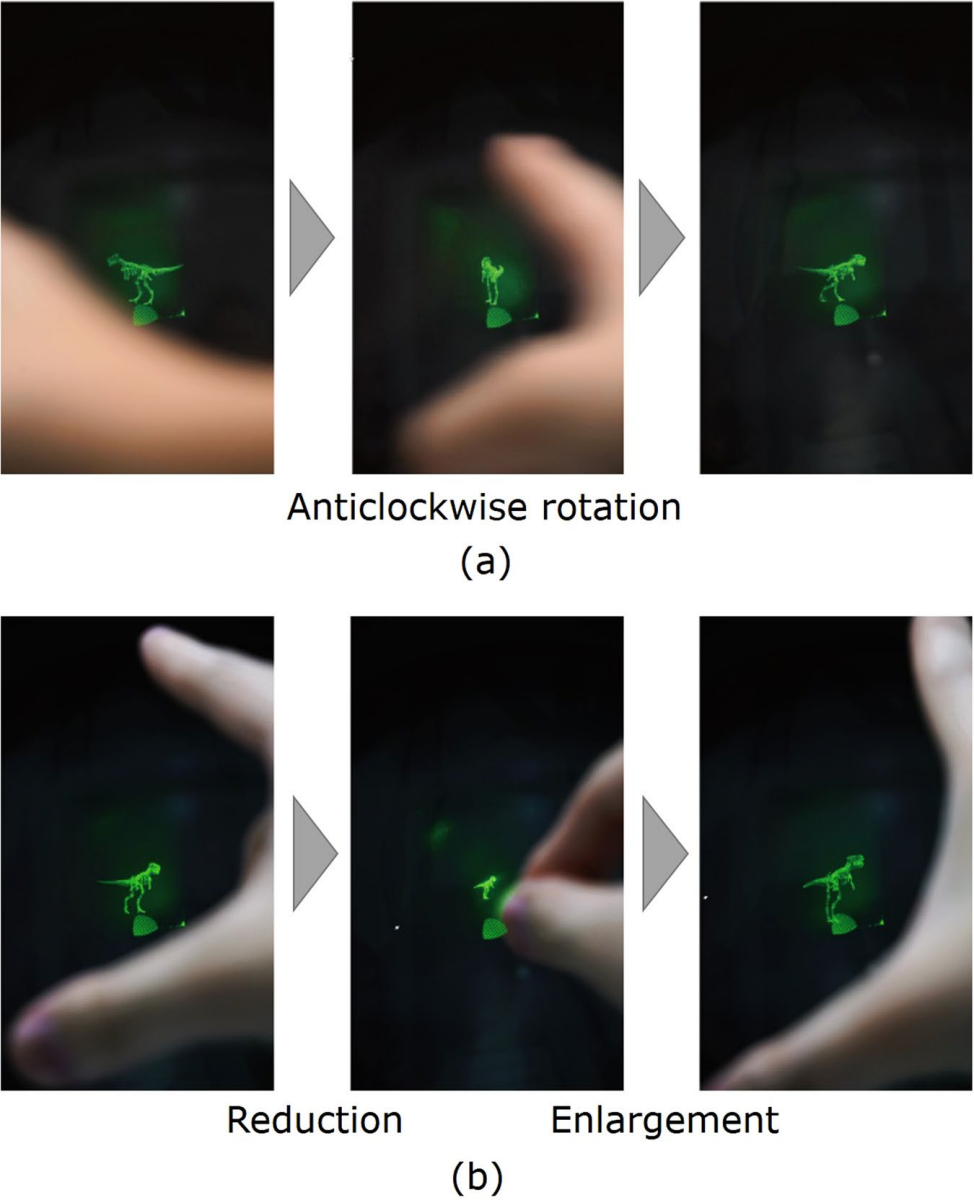
Images showing an operator (**a**) rotating the image using a swiping gesture and (**b**) reducing and enlarging the image using pinching gestures.

Figure 4 and supplementary movie M1 show the reconstructed images acquired using the constructed system. Figure 4a shows the reconstructed image being rotated by the observer using a swiping gesture. Figure 4b,c show the image being enlarged and reduced by the observer using pinching gestures, respectively. The average calculation time of the system was 27.6 ms per CGH, corresponding to 36.2 fps. Using only the CPU, the average calculation time was 1.39 s; thus, the parallelised system with both the GPU and CPU was 50.4 times faster than the system based on the CPU alone.

As shown above, we successfully demonstrated that an observer can interactively handle the reconstructed image in real time using swiping and pinching gestures. The new interactive finger-sensitive system enables an observer to intuitively handle electro-holographic images in real time using finger gestures.

## Discussion

To reconstruct more realistic 3D images close to an actual object, a system capable of full-colour reconstruction with red and blue light in addition to green light is needed. Thus, we extended the single-colour system to a full-colour reconstruction system to allow interaction with more realistic 3D images.

**Figure 5:**
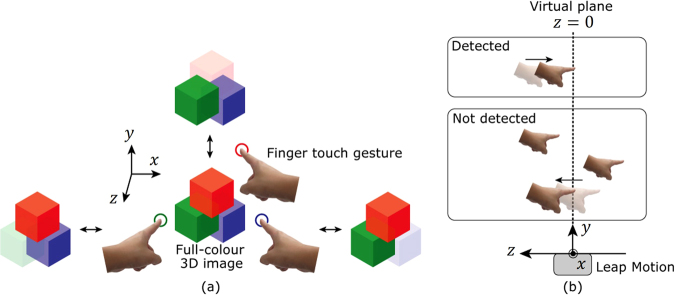
Schematics of (**a**) the full-colour interactive image reconstruction system using finger touch gestures and (**b**) detection of the finger touch gestures using the Leap Motion sensor.

Figure 5a shows a schematic of the full-colour interactive image reconstruction system. Using the single-colour interactive system described in the Results section, we only detected the swiping and pinching gestures. This indicates that we did not use the depth information detected by the motion sensor. We then implemented depth detection in the full-colour interactive image reconstruction system. This system detects finger touch gestures using a motion sensor and switches between the display and non-display of the red, green and blue components depending on the position of the finger gesture. Here, we set a virtual plane over the Leap Motion sensor and calibrated the depth position of the virtual plane as *z* = 0, as shown in Fig. 5b. The motion sensor detects the *z*-coordinate as well as the direction of the movement of the observer’s finger position along the *z*-axis. The motion sensor regards the finger motion as the touch gesture only when the finger moves from the positive range (*z* > 0) to the negative range (*z* < 0). Additionally, the motion sensor detects the x- and y-coordinates on the virtual plane simultaneously. When the touch position is above the full-colour reconstructed image, the system switches to the red reconstructed image. When the position is to the left of the image, the system switches to the green reconstructed image. Finally, when the touch position is to the right, the system switches to the blue reconstructed image. In all cases, only the colour corresponding to the finger touch position changes; the other colours remain the same.

**Figure 6:**
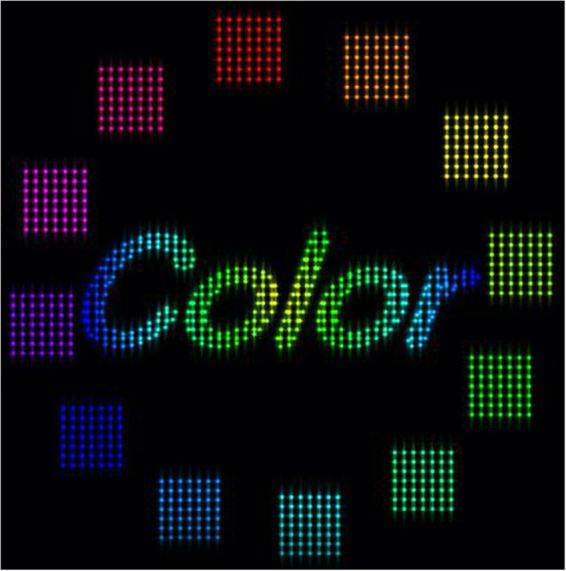
The object used in the demonstration of the full-colour interactive system.

The experimental setup used for full-colour reconstruction is shown in Fig. 3. In addition to the green laser and one SLM for the green reconstructed image, we used a red laser (650 nm), a blue laser (450 nm) and two additional SLMs to display red and blue reconstructed images. The 3D object used in the demonstration (Fig. 6) consisted of an average of 1,709 point light sources for all frames. The full-colour object was virtually placed 1 m away from the SLMs when calculating the CGHs.

**Figure 7:**
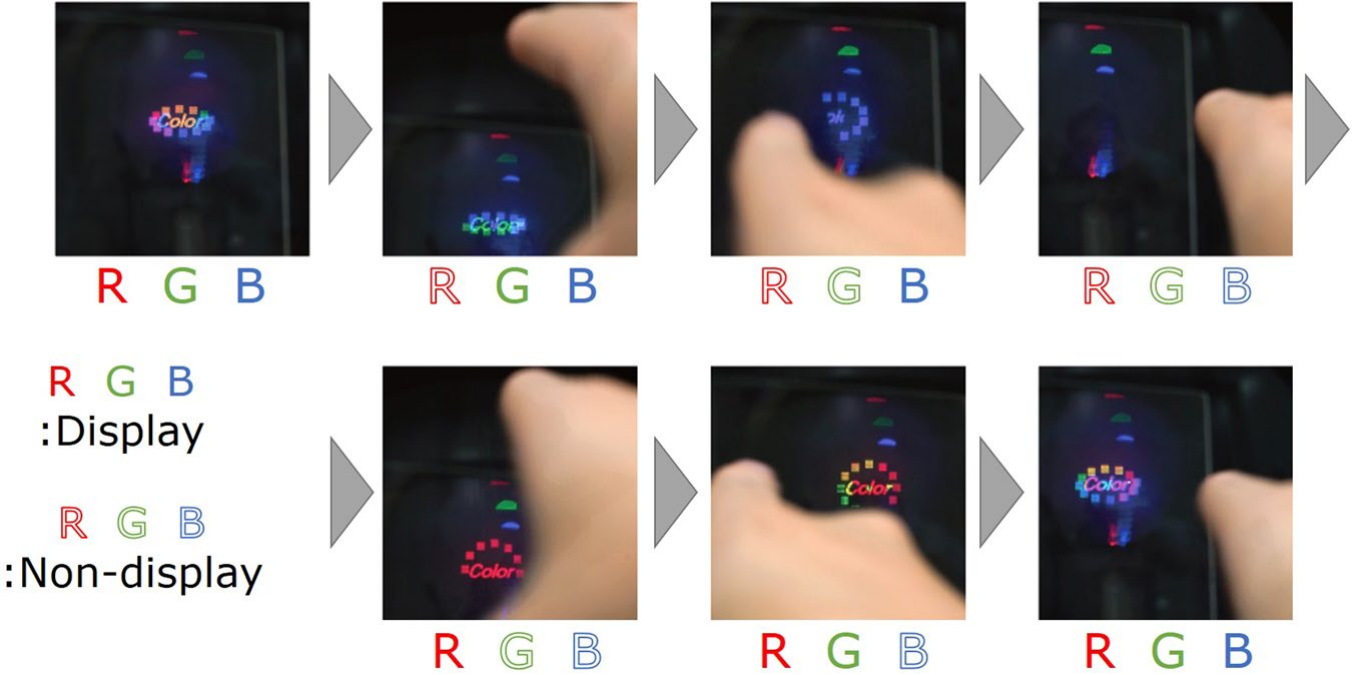
Images showing an operator switching the display of a full-colour 3D image using finger touch gestures.

Figure 7 and supplementary movie M2 show an operator switching between display and non-display full-colour images by touching the display. By touching the display to the top, left and right of the full-colour reconstructed image, the full-colour interactive system switched between display and non-display of the red, green and blue reconstructed images, respectively. The average calculation time of the system was 22.6 ms per CGH; thus, the system displayed the full-colour reconstructed image at more than 40 fps. The successful demonstration indicates that the developed system enables the interactive handling of full-colour reconstructed images using finger touch gestures.

In both the single-colour and full-colour reconstructed system, it is difficult to reconstruct and interactively handle more realistic and high-definition 3D images (e.g. a 3D image comprising tens of thousands of point light sources) in real time. Hence, it is necessary to speed up the CGH calculation in order to reconstruct 3D images composed of more point light sources. The computational complexity of a CGH is *O*(*LN*_*x*_*N*_*Y*_), where *L* is the number of point light sources, *N*_*x*_ is the number of pixels of the CGH along the horizontal direction and *N*_*Y*_ is the number of pixels along the vertical direction.

One method used to speed up the CGH calculation is to distribute the calculation load over multiple GPUs^33,34^. For instance, a full-colour interactive handling system with three GPUs that distributes the CGH calculation of the each colour to one of the three GPUs will be able to calculate the CGH three times faster than the system demonstrated in this study with only one GPU. Using this method allows high-definition images with large numbers of point light sources to be reconstructed. Another method for speeding up the CGH calculation is to adopt a fast calculation algorithm such as wavelet shrinkage-based superposition (WASABI)^35^. The speed enhancement achieved by WASABI depends on the selectivity rate *S* of the representative wavelet coefficients. Even if *S* is reduced to 1%, WASABI can obtain almost the same reconstructed-image quality as that obtained without WASABI. In this case, WASABI accelerates CGH calculation by 100 times compared to the method with no fast calculation algorithm.

## Methods

### CGH calculation

**Figure 8:**
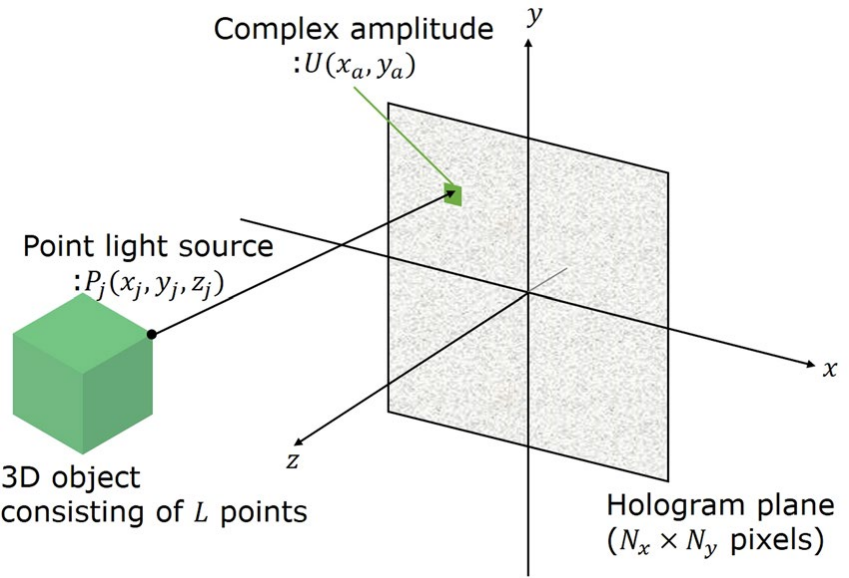
Schematic showing the method used to calculate CGH.

Figure 8 shows a schematic of CGH calculation, which is expressed as follows:

1 $$U({x}_{a},{y}_{a})=\sum _{j=1}^{L}{A}_{j}\,\exp \{-i\frac{2\pi }{\lambda }\frac{{({x}_{a}-{x}_{j})}^{2}+{({y}_{a}-{y}_{j})}^{2}}{2{z}_{j}}\},$$

where (*x*_*a*_, *y*_*a*_) are the coordinates on the CGH, (*x*_*j*_, *y*_*j*_, *z*_*j*_) are the coordinates of the *j*-th point light source, *L* is the number of point light sources, *i* is an imaginary unit, *λ* is the wavelength of light, *A*_*j*_ is the field magnitude emitted from a point light source and *U* is the complex amplitude calculated by point light sources and the coordinates of the CGH. Because we used a phase modulation-type SLM, we calculated a phase modulation-type CGH pattern,*ϕ*(*x*_*a*_, *y*_*a*_), using the following equation:

2 $$\varphi ({x}_{a},{y}_{a})={\rm{\arg }}(U({x}_{a},{y}_{a})),$$

where arg(*U*) is an operator indicating the argument of a complex number.

For a full-colour CGH, the wavelength in Eq. (1) is set to the red, green or blue wavelength and the CGH of each colour is calculated. Thus, the calculation time for the full-colour CGH is three times greater than that of the monochrome one. When constructing a full-colour optical system, red, green and blue CGHs can be displayed on one SLM^36–39^ or on three different SLMs^40^. For the one-SLM system, there are several methods based on time-division multiplexing^36^, space-division multiplexing^37^, superposition of the red, green and blue images on a diffuser plate^38^ and the use of SLMs with high dynamic phase modulation range^39^. Time-division multiplexing requires a high-speed SLM, such as a digital micro-mirror device, to obtain full-colour images without flickers caused by the switching of the reference lights to other colours. Space-division multiplexing decreases the resolution of each colour hologram because this method displays each colour hologram on the different regions of an SLM plane. A superposition method is proposed for a holographic projector^41,42^. This method is not suitable for the reconstruction of 3D images because it projects holographic images using a 2D diffuser plate. The last method requires a special phase-only SLM, which has high dynamic phase modulation range (i.e. 0–10π). Because the SLM used herein has the phase modulation range of 0–2π and a refresh rate of 60 Hz, we adopted the method using of three different SLMs.

To realise interactive handling without discomfort, the system requires real-time operation, which is difficult to achieve using only a CPU. Hence, we parallelised the processing of point clouds and the calculation of CGH by combining a CPU with a GPU.

### Parallelisation of the CGH calculation

Since Eqs (1) and (2) are solved independently for each pixel, the system assigns one pixel calculation to one thread using the GPU. After the CGH calculation is complete for all pixels, the system sends the CGH result to the CPU. To calculate the full-colour CGH, the system solves Eqs (1) and (2) in the order of blue, green and red wavelengths within each thread. Finally, the system sends the full-colour CGH to the CPU.

## Electronic supplementary material

movie M1

movie M2
